# Drug-related problems in type 2 diabetes mellitus patients with dyslipidemia

**DOI:** 10.1186/1471-2458-13-1192

**Published:** 2013-12-17

**Authors:** Hasniza Zaman Huri, Lee Chai Ling

**Affiliations:** 1 Department of Pharmacy, Faculty of Medicine, University of Malaya, Kuala Lumpur 50603, Malaysia; 2 Clinical Investigation Centre, University Malaya Medical Centre, 13th Floor Main Tower, Lembah Pantai, Kuala Lumpur 59100, Malaysia

**Keywords:** Type 2 diabetes mellitus, Drug-related problems, Dyslipidemia

## Abstract

**Background:**

Drug-Related Problems (DRPs) commonly occur among type 2 diabetes mellitus (T2DM) patients. However, few studies have been performed on T2DM patients with dyslipidemia. This purpose of this study was to assess drug-related problems (DRPs) and factors associated with its occurrence.

**Methods:**

The retrospective study involved 208 T2DM in-patients and out-patients with dyslipidemia, and was conducted at a tertiary hospital in Malaysia from January 2009 to December 2011. The identification and assessment of DRPs were based on the Pharmaceutical Care Network Europe (PCNE) tool version 5.01. The potentially inappropriate medication use in older adults was assessed based on the American Geriatrics Society updated Beers Criteria.

**Results:**

A total of 406 DRPs were identified. Among these patients, 91.8% had at least one DRP, averaging 1.94 ± 1.10 problems per patient. The majority of T2DM patients with dyslipidemia (91.8%) had at least one DRP. The most frequent types of DRP were potential drug-drug interaction (18.0%), drug not taken or administered (14.3%) and insufficient awareness of health and diseases (11.8%). Anti-hypertensive, lipid-modifying and anti-diabetic agents were the drug classes that were most likely to be associated with DRPs. Male gender, renal impairment, polypharmacy and poor lipid control were factors that were significantly associated with DRP in diabetic dyslipidemia patients.

**Conclusion:**

Early identification of DRPs and factors associated with them are essential to prevent and resolve DRPs in T2DM patients with dyslipidemia.

## Background

Dyslipidemia is a common co-morbidity in T2DM patients [1]. According to the Center for Disease Control and Prevention (CDC), 70% to 97% of T2DM adults have one or more lipid abnormalities [2]. In T2DM patients, dyslipidemia is characterized by an elevated triglyceride (TG) level, a decrease in high density lipoprotein cholesterol (HDL-C) level and the presence of smaller and denser low density lipoprotein cholesterol (LDL-C) particles [1,3].

Dyslipidemia in T2DM is associated with an increased flux of free fatty acid release from insulin-resistant fat cells to the liver. Consequently, the deposition of lipid in blood vessels causes atherosclerotic lesions which later lead to cardiovascular disease (CVD) [3]. In addition, the risk of coronary artery disease (CAD) in T2DM patients increases 2- to 4-fold compared to non-diabetic patients [4]. The co-existence of dyslipidemia in T2DM patients will further increase the risk of developing CAD [3,4].

Drug-related problems (DRPs) are pharmacotherapy problems that actually or potentially have an impact on desired health outcome [5]. There is a high prevalence of DRP in T2DM patients, in which an average of about 4 DRPs occurred in a patient [6,7]. This is probably due to patients receiving multiple drugs to control their medical conditions, all of which promote DRPs. Several factors could contribute to DRPs. For instance, liver or renal impairment causes DRP via the alteration of the pharmacokinetics of anti-diabetic and lipid-modifying agents [8,9]. In geriatrics, co-morbidities, poor medication adherence and polypharmacy potentially cause DRPs [10,11].

Dyslipidemia and T2DM contribute substantially to cardiovascular complications [3,4]; hence the optimization of management by the identification and prevention of DRPs is essential. There is a lack of studies on DRPs in T2DM patients with dyslipidemia, both locally and globally. Therefore, this study aims to investigate DRPs in T2DM patients with dyslipidemia and its objectives are to assess drug treatment use, DRPs and factors affecting DRPs in T2DM patients with dyslipidemia. The findings from this study can help to determine the pattern of DRPs in this population and be used as preliminary data for future studies.

## Methods

### Study population and sampling frame

The study population consisted of all T2DM in-patients and out-patients with dyslipidemia identified between January 2009 and December 2011. The sample size was calculated based on the Epi Info Program version 7.0 (CDC, Clifton Rd. Atlanta, USA) with a minimum of 196 patients to give a power of β = 0.8 and a confidence level of 95%.

### Study design and procedures

**Figure 1:**
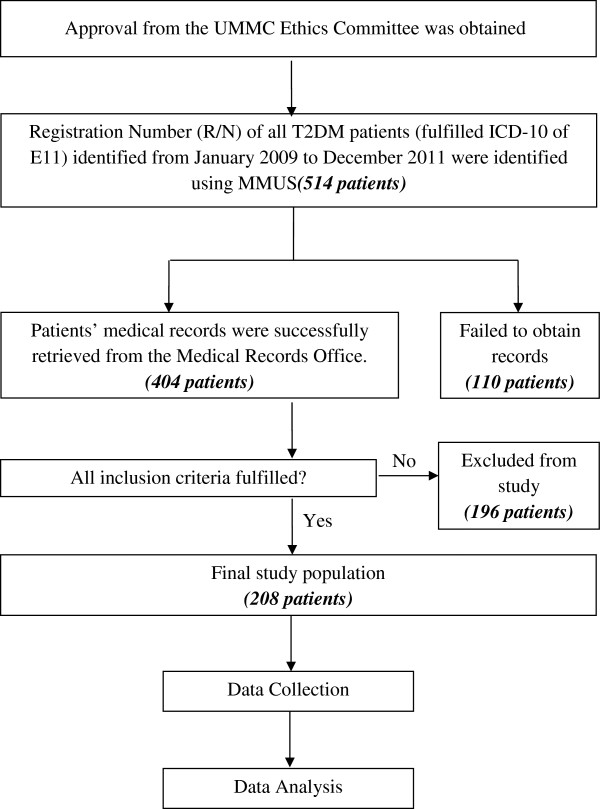
Overview of study procedure.

The retrospective study was conducted in a tertiary teaching hospital, namely the University of Malaya Medical Centre (UMMC), Malaysia following approval by the UMMC Ethics Committee. A total of 208 patients who fulfilled the inclusion criteria (Table 1) were included in this study (Figure 1). Table 2 shows the definitions that were used in this study.

**Table 1:** Inclusion and exclusion criteria of the study

***Inclusion criteria:***	
Adult patient above 18 years old and	
(1)	Diagnosed with T2DM and prescribed with at least one anti-diabetic drug.
(2)	Diagnosed with dyslipidemia and prescribed at least one LLA
***Exclusion criteria:***	
(1)	Patient with a disease other than T2DM.
(2)	Diagnosed with dyslipidemia but not on any pharmacological treatment.
(3)	Patient prescribed with LLA but not diagnosed with any lipid disorders.
(4)	Patient with incomplete data

**Table 2:** Definition of terms used in the study

**Characteristics**	**Definition**	**References**
**Demographic**	*1) Elderly*	[12]
	- Older adults above 64 years old	
**Co-morbidities**	*1) Cardiovascular accident (CVA)*	[1,13]
	- Refers to stroke, transient ischemic attack and hemorrhagic stroke, ischemic heart disease (IHD)	
	*2) Liver impairment*	
	- Refers to chronic hepatitis, liver cirrhosis, fatty liver, elevation of liver enzyme such as alanine transaminase (ALT) and aspartate transaminase (AST) of above 3 times upper limit of normal level	
	*3) Renal impairment*	
	- Creatinine clearance of <60 ml/min or as stated in medical records.	
**Microvascular complications**	*1) Retinopathy*	[1]
	- Refers to funduscopic deterioration or as stated in medical records.	
	*2) Neuropathy*	
	- For sensory, included history of foot lesions; for autonomic, included sexual dysfunction and gastroparesis or as stated in medical records.	
**Metabolic control**	*1) Glycemic control*	[1,14-16]
	- Good glycemic control refers to achieving targeted level of A1C <6.5%.	
	*2) Lipid control*	
	- Good lipid control refers to achieving all lipid fractions targeted level in which LDL-C <2.6 mmol/L, TG <1.7 mmol/L and HDL-C >1.0 mmol/L.	
	- Poor lipid control refers to those patients unable to achieve one of the lipid fractions within targeted range.	
**Drug therapy**	*1) Polypharmacy*	[17]
	- Six or more chronic medications for duration of at least one month.	

### Identification of DRPs

The PCNE tool version 5.01 [5] was used to identify and assess DRPs in this study. The assessment of DRPs was based on each researcher’s clinical judgment with the support of established literature and standard guidelines of diseases [16,18-20].

Information on drugs, such as recommended dosages, frequency, potential interactions and side-effects, was based on the Drug Information Handbook [21] and the British National Formulary [22]. The potentially inappropriate medication use in older adults was assessed based on the American Geriatrics Society updated Beers Criteria [12]. Table 3 and 4 shows definitions for DRPs and causes associated with DRPs respectively.

**Table 3:** Definition of DRP classification

**Code**	**DRP**	**Definition**
P1	Adverse reactions	Patient suffers from an adverse drug event
P2	Drug choice problem	Patient gets or is going to get an incorrect (or no drug) drug for disease or condition
P3	Dosing problem	Patient gets more or less than the amount of drug he or she requires
P4	Drug use problem	1) Incorrect drug taken by patient
		2) No drug taken/administered for at least one dose of total daily dose
P5	Interactions	There is a manifest or potential drug-drug or drug-food or drug-disease interaction

Adapted from: [5,22].

**Table 4:** Definition of causes associated with DRP

**Code**	**Primary domain**	**Code**	**Cause associated with DRP**
**C1**	**Drug/Dose selection**	**C1.1**	Inappropriate drug selection
	The cause of the DRP is related to the selection of the drug and/or dosage schedule		
		**C1.2**	Inappropriate dosage selection
		**C1.3**	More cost effective drugs available
		**C1.4**	Pharmacokinetic problems, incl. ageing/deterioration in organ function and interactions
		**C1.5**	Synergistic/preventive drug required and not given
		**C1.6**	Deterioration/improvement of disease state
		**C1.7**	New symptom or indication revealed/presented
		**C1.8**	Manifest side effect, no other cause
**C2**	**Drug use process**	**C2.1**	Inappropriate timing of administration and/or dosing intervals
	The cause of the DRP can be related to the way the patient uses the drug, in spite of proper dosage instructions (on the label)		
		**C2.2**	Drug underused/under-administered
		**C2.3**	Drug overused/over-administered
		**C2.4**	Therapeutic drug level not monitored
		**C2.5**	Drug abused (unregulated overuse)
		**C2.6**	Patient unable to use drug/form as directed
**C3**	**Information**	**C3.1**	Instructions for use/taking not known
	The cause of the DRP can be related to a lack or misinterpretation of information		
		**C3.2**	Patient unaware of reason for drug treatment
		**C3.3**	Patients has difficulties reading patient information form/leaflet
		**C3.4**	Patient unable to understand local language
		**C3.5**	Lack of communication between healthcare professionals
**C4**	**Patient/Psychological**	**C4.1**	Patient forgets to use/take drug
	The cause of the DRP can be related to the personality or behavior of the patient.		
		**C4.2**	Patient has concerns with drugs
		**C4.3**	Patent suspects side-effect
		**C4.4**	Patient unwilling to carry financial costs
		**C4.5**	Patient unwilling to bother physician
		**C4.6**	Patient unwilling to change drugs
		**C4.7**	Patient unwilling to adapt life-style
		**C4.8**	Burden of therapy
		**C4.9**	Treatment not in line with health beliefs
		**C4.10**	Patient takes food that interacts with drugs
**C5**	**Logistics**	**C5.1**	Prescribed drug not available (anymore)
	The cause of the DRP can be related to the logistics of the prescribing or dispensing mechanism		
		**C5.2**	Prescribing error (only in case of slip of the pen)
		**C5.3**	Dispensing error (wrong drug or dose dispensed)
		**C5.1**	Prescribed drug not available (anymore)
**C6**	**Others**	**C6.1**	Other cause, specify
		**C6.2**	No obvious cause
**Total**			

Adapted from: [5,22].

### Data analysis

All extracted data were pooled and analyzed using the Statistical Package for the Social Sciences (SPSS) software version 20.0 (SSPS Inc., Chicago, IL, USA). Continuous data, such as A1C values and lipid profiles, were tested for normality using the Kolmogorov-Smirnov test. A normally distributed result was expressed as mean ± standard deviation whilst the non-symmetrically distributed data was presented as a median with the minimum and maximum value. For categorical data, the Chi-squared test was used to determine the association of patient’s characteristics and the occurrence of DRP. When the expected cell count for >20% was less than 5, Fisher’s Exact Test was used. On the other hand, the *T*-test was used to compare mean between groups for continuous data. The statistical significance was assumed at p < 0.05 in this study. The summarized findings were rearranged and tabulated in a graphical or table form.

## Results

### Demographic characteristics

A total of 208 T2DM patients fulfilled the inclusion criteria in this study. Males (53.8%) are present in an almost equal proportion as female patients in this study. The non-elderly group of patients (56.7%) was slightly larger than the elderly group. The mean ± standard deviation (SD) of patients’ age was 61.7 ± 13.3 years old with the minimum and maximum ages of 23 and 96 years old, respectively.

### Clinical characteristics

The majority of the patient population had duration of T2DM of less than or equal to 10 years (n = 137), and most of the patients in this study were non-smokers (66.4%). Results also showed that about 1 in 10 subjects consumed alcohol (9.6%). Besides that, more than 70% of subjects were found to have polypharmacy.

Hypertension contributed to the highest percentage among all of the categories of co-morbidities. About a quarter of renal impairment subjects (25.3%) were on renal replacement therapy. There were only 5.8% (or 12 subjects) without any co-morbidity.

### Metabolic control

There were only 49 subjects’ (23.6%) with an A1C value within the targeted range (less than 6.5%). In this study, the A1C values were not normally distributed. Hence, result was presented as median and range. Mean of A1C value is 8.72 ± 0.19% in this study population. All of the lipid parameters such as total cholesterol, LDL-C, HDL-C and triglyceride were not normally distributed when tested with normality test (refer Table 5).

**Table 5:** Metabolic control of patients (n = 208)

**Characteristics**	*Median	**Range**	
		**Minimum value**	**Maximum value**
**A1C (%)**	7.80	4.90	17.40
**Lipid profile (mmol/L)**			
Total cholesterol	4.70	1.25	9.30
LDL-C*	2.68	0.27	7.34
HDL-C	1.08	0.02	2.52
Triglyceride	1.80	0.50	34.6

*LDL-C level was not calculated when TG ≥4.5 mmol/L based on the UMMC’s Laboratory Information System (LIS).* Results were not normally distributed.

**Figure 2:**
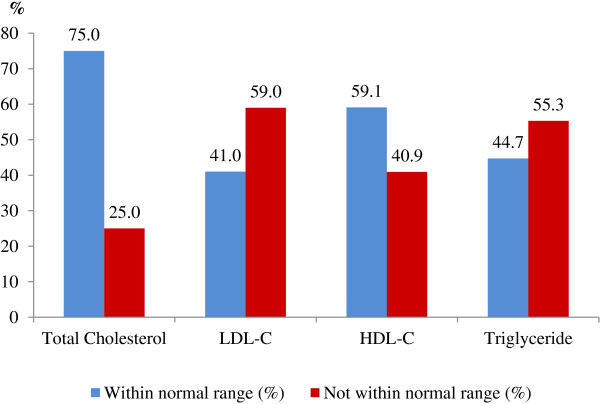
Lipid profiles of T2DM patients with dyslipidemia.

About a quarter (24.5%) of the subjects had all lipid parameters within the recommended range. The remaining subjects had at least one lipid parameter that was not within the normal range. Figure 2 shows the lipid profiles of the subjects. Most of the patients were unable to achieve the LDL-C and TG targeted levels, which were <2.6 mmol/L and <1.7 mmol/L, respectively.

### Medication used in T2DM patients with dyslipidemia

The percentages of patients prescribed with combination insulin therapy (24.5%), combination insulin and oral hypoglycemic (OHA) therapy (24.0%), combination OHA therapy (23.1%) and monotherapy of OHA (22.1%) were about the same. Among the subjects, only 13 received monotherapy of insulin. The most common anti-diabetic drug prescribed in the study was metformin either as a monotherapy or combination therapy (28.5%).

On the other hand, the most common lipid lowering agent (LLA) prescribed in subjects was simvastatin (151 subjects or 72.6%). There was only one subject (0.5%) prescribed either rosuvastatin or pravastatin. The mean number of chronic medications prescribed per patient was 4.8 ± 3.3 medications. The two leading concurrent chronic medications were anti-platelet (65.4%) and ACE inhibitor (55.8%). Nevertheless, 11 subjects (5.3%) were not on any medication except for anti-diabetic agents and LLA.

### Drug-related problems

A total of 91.8% of patients (n = 191) had at least one DRP. A total of 406 DRPs were identified and the mean number of DRPs per patient was 1.94 ± 1.10. Out of 6 domains of DRP, 18 categories had at least one problem reported. The identified DRPs were widely distributed, with the top three categories being “potential interaction”, “drug not taken or administered at all” and “insufficient awareness of health and diseases”. Table 6 reports the detailed classifications of DRPs in 191 subjects.

**Table 6:** Classification of DRP (N = 191)

**Code**	**Detailed classification**	**n (%)**
**P1**	**Adverse reactions**	**31 (7.6)**
P1.1	Side-effect suffered (non-allergic)	27 (6.6)
P1.2	Side-effect suffered (allergic)	4 (1.0)
**P2**	**Drug choice problem**	**106 (26.1)**
P2.1	Inappropriate drug (not most appropriate for indication)	27 (6.7)
P2.2	Inappropriate drug form (not most appropriate for indication)	1 (0.2)
P2.3	Inappropriate duplication of therapeutic group or active ingredient	9 (2.2)
P2.4	Contra-indication for drug (incl. Pregnancy/breast feeding)	15 (3.7)
P2.5	No clear indication for drug use	8 (2.0)
P2.6	No drug prescribed but clear indication	46 (11.3)
**P3**	**Dosing problem**	**58 (14.3)**
P3.1	Drug dose too low or dosage regime not frequent enough	41 (10.1)
P3.2	Drug dose too high or dosage regime too frequent	10 (2.5)
P3.3	Duration of treatment too short	6 (1.5)
P3.4	Duration of treatment too long	1 (0.2)
**P4**	**Drug use problem**	**58 (14.3)**
P4.1	Drug not taken/administered at all	58 (14.3)
**P5**	**Interactions**	**73 (18.0)**
P5.1	Potential interaction	73 (18.0)
**P6**	**Others**	**80 (19.7)**
P6.1	Patient dissatisfied with therapy despite taking drug(s) correctly	23 (5.7)
P6.2	Insufficient awareness of health and diseases (possibly leading to future problems)	48 (11.8)
P6.3	Unclear complaints. Further clarification necessary	2 (0.5)
P6.4	Therapy failure (reason unknown)	7 (1.7)
**Total**		*406 (100.0)

*A patient may have one or more DRP. DRPs of code P1.3 (toxic effect suffered), P4.2 (wrong drug taken/administered at all) and P5.2 (manifest interaction) were not listed in the table as none of the patients experienced these problems.

The independent *T*-test showed that patients with DRPs have significantly higher A1C values than those without any DRPs (8.8% versus 7.4%, p = 0.004). However, there was no significant difference between DRPs and the mean of lipid fractions, which included total cholesterol (p = 0.247), LDL-C (p = 0.560), HDL-C (p = 0.092) and triglycerides (p = 0.338).

A total of 491 causes associated with DRPs were identified among the 34 categories of PCNE classifications. The mean number of causes of DRP per patient was 2.37 ± 1.40. The three leading causes were “pharmacokinetic problems”, “inappropriate dosage selection” and “synergistic or preventive drug required and not given”. Table 7 shows the causes associated with DRP in 191 subjects.

**Table 7:** Causes associated with DRP (N = 191)

**Code**	**Detailed classification**	**n (%)**
**C1**	**Drug/Dose selection**	**277 (56.5)**
**C1.1**	Inappropriate drug selection	36 (7.3)
**C1.2**	Inappropriate dosage selection	69 (14.1)
**C1.4**	Pharmacokinetic problems, incl. ageing/deterioration in organ function and interactions	84 (17.1)
**C1.5**	Synergistic/preventive drug required and not given	48 (9.8)
**C1.6**	Deterioration/improvement of disease state	11 (2.2)
**C1.7**	New symptom or indication revealed/presented	12 (2.4)
**C1.8**	Manifest side effect, no other cause	17 (3.6)
**C2**	**Drug use process**	**22 (4.4)**
**C2.1**	Inappropriate timing of administration and/or dosing intervals	6 (1.2)
**C2.2**	Drug underused/under-administered	5 (1.0)
**C2.3**	Drug overused/over-administered	3 (0.6)
**C2.4**	Therapeutic drug level not monitored	3 (0.6)
**C2.5**	Drug abused (unregulated overuse)	3 (0.6)
**C2.6**	Patient unable to use drug/form as directed	2 (0.4)
**C3**	**Information**	**31 (6.4)**
**C3.1**	Instructions for use/taking not known	17 (3.6)
**C3.2**	Patient unaware of reason for drug treatment	5 (1.0)
**C3.4**	Patient unable to understand local language	7 (1.4)
**C3.5**	Lack of communication between healthcare professionals	2 (0.4)
**C4**	**Patient/Psychological**	**140 (28.5)**
**C4.1**	Patient forgets to use/take drug	36 (7.3)
**C4.2**	Patient has concerns with drugs	11 (2.2)
**C4.3**	Patent suspects side-effect	3 (0.6)
**C4.4**	Patient unwilling to carry financial costs	8 (1.6)
**C4.5**	Patient unwilling to bother physician	14 (2.9)
**C4.6**	Patient unwilling to change drugs	1 (0.2)
**C4.7**	Patient unwilling to adapt life-style	45 (9.2)
**C4.8**	Burden of therapy	1 (0.2)
**C4.9**	Treatment not in line with health beliefs	19 (3.9)
**C4.10**	Patient takes food that interacts with drugs	2 (0.4)
**C5**	**Logistics**	**18 (3.6)**
**C5.1**	Prescribed drug not available (anymore)	8 (1.6)
**C5.2**	Prescribing error (only in case of slip of the pen)	8 (1.6)
**C5.3**	Dispensing error (wrong drug or dose dispensed)	2 (0.4)
**C6**	**Others**	**3 (0.6)**
**C6.2**	No obvious cause	3 (0.6)
**Total**		*491 (100.0)

*A DRP associated with one or more causes. Causes of code C1.3 (More cost-effective drug available), C3.3 (Patient has difficulties reading/understanding Patient Information Form/Leaflet) and C6.1 (Other cause) were not listed in the table as none of the DRPs were associated with these causes.

**Figure 3:**
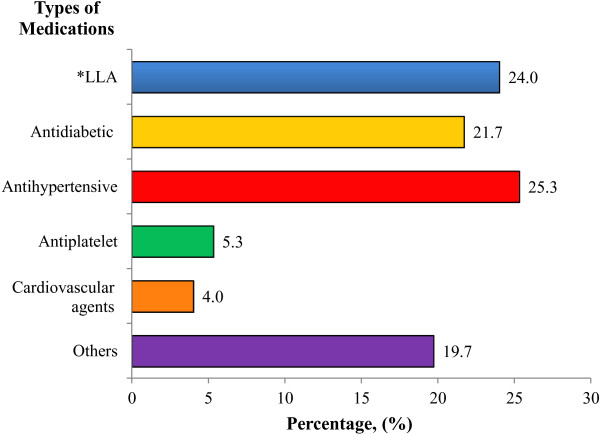
Medication categories that causes drug-related problems; * LLA refers to lipid-lowering agents.

There were 304 drugs that caused DRPs in subjects. The drug class that was most likely to cause DRPs was antihypertensive agents. This was followed by lipid-lowering agents and anti-diabetic agents. Examples of other medications include as warfarin, iron supplements and glyceryl trinitrate. Different medication categories that caused DRPs are shown in Figure 3.

### Factors that were significantly associated with DRPs in T2DM patients with dyslipidemia

Four factors (male gender, renal impairment, polypharmacy and lipid profile) were found to be significantly associated with DRPs in T2DM patients with dyslipidemia (p <0.05). Males had more DRPs compared to females. In addition, patients suffering from renal impairment had a higher probability of having at least one DRP compared to patients with normal renal function.

There was also a significant difference in the patients having polypharmacy compared to patients without polypharmacy. Patients that were presented with more than six drugs were at increased risk of the occurrence of DRPs. On the other hand, patients with at least one lipid parameter not within the targeted range had a higher occurrence of DRP. Table 8 summarizes the factors that were significantly associated with DRP.

**Table 8:** Factors significantly associated with DRP (n = 191)

**Characteristics**	**Chi squared**	*DRP		^**δ**^**p Value**
		**Yes**	**No**	
**Gender**				
Male	5.583	108 (96.4%)	4 (3.6%)	0.011
Female		83 (86.5%)	13 (13.5%)	
**Renal impairment**				
Yes	5.596	85 (97.7%)	2 (2.3%)	0.009
No		106 (87.6%)	15 (12.4%)	
**Polypharmacy**				
Yes	4.264	141 (94.6%)	8 (5.4%)	0.039
No		50 (84.7%)	9 (15.3%)	
**Lipid control**				
Good lipid control	9.840	41 (80.4%)	10 (19.6%)	0.002
Poor lipid control		150 (95.5%)	7 (4.5%)	

*DRP reported as the number of patients (percentage across row,%); ^δ^degrees of freedom = 1.

### Factors that were not significantly associated with DRP in T2DM patients with dyslipidemia

Factors that were not significantly (p > 0.05) associated with DRP in T2DM patients with dyslipidemia are shown in Table 9.

**Table 9:** Parameters that were not significantly associated with DRP

**Characteristics**	**n**	**Number of patients (percentage,%)**	**p Value**
**Age**			
Non-elderly (18-64 years old)	208	118 (56.7)	0.662^b^
Elderly (≥65 years old)		90 (43.3)	
**Ethnicity**			
Malay	208	90 (43.3)	
Chinese		38 (18.3)	0.934^a^
Indian		76 (36.5)	
Others		4 (1.9)	
**Duration of T2DM**			
≤10 years	137	79 (57.7)	
11-20 years		30 (21.9)	0.442^a^
21-30 years		20 (14.6)	
≥31 years		8 (5.8)	
**Smoking habit**			
Smoker	200	37 (18.5)	0.496^a^
Non-smoker		138 (69.0)	
Ex-smoker		25 (12.5)	
**Alcohol consumption**			
Yes	194	20 (10.3)	0.584^a^
No		161 (83.0)	
Ex-drinker		13 (6.7)	
**Complications**			
Neuropathy	208	19 (9.1)	0.192^c^
Retinopathy	208	50 (24.0)	>0.999^c^
**Co-morbidities**			
Hypertension	208	167 (80.3)	0.465^b^
CVA	208	41 (19.7)	>0.999^c^
IHD	208	69 (33.2)	0.188^c^
Liver impairment	208	23 (11.1)	>0.999^c^
Thyroid disorder	208	10 (4.8)	>0.999^c^
Others	208	87 (41.8)	>0.999^b^
**Glucose control**			
A1C less than 6.5%	208	49 (23.6%)	0.137^b^
A1C more or equal than 6.5%		159 (76.4%)	

^a^Pearson Chi Squared Test; ^b^Continuity Correction; ^c^Fisher Exact Test.

## Discussion

### Drug-related problems

The PCNE classification that was used in this study has been critically appraised as the most appropriate classification that reflects outcomes, and the results are reproducible [23,24]. The classification tool has been validated and was used in many other published studies to assess DRP occurrence [10,24-26].

In this study, the mean of 2 DRPs per patient was less than the levels found in previous studies, which were about 4 DRPs per T2DM patient [6,7]. This could be explained by the different DRP classification tool used, which is more general compared to the PCNE tool. On the other hand, the prevalence was high, with at least 9 out of 10 T2DM patients with dyslipidemia having problems with drugs in this study. A study by Bob & Ines [7] which used the same classification tool showed that all T2DM patients had at least 1 DRP. The result was significantly different as the methodology of the studies was not the same. The assessment of DRP in the current study solely depended on the review of medical and biochemistry records. Meanwhile, Bob & Ines [7] instilled a qualitative interview method in their research to identify DRPs in T2DM patients. However, the high occurrence of DRPs in this population of patients shows that there was lack of optimal pharmacologic management in clinical practice.

The two most common DRP classifications identified in the current study were “potential interaction” and “drug not taken or administered at all”. These findings were not in line with previous studies in T2DM populations conducted by Anne *et al.*[27] and Haugbolle *et al.*[6]. Both of the studies reported “adverse drug reaction” and “inappropriate use of medicines by the patients” as the most common DRPs, respectively [6,27]. The frequency of various drug categories varied among studies as this depends on the methodology (such as medical review or interview technique) and types of DRP classification (such as PCNE or PI-Doc system) used.

### Potential interaction

The high number of medications used and the combination of various drug classes contributed to the high prevalence of significant potential drug-drug interactions or drug-disease interactions in this population (17.5%). This result was consistent with a study by Bob & Ines [7] in which about 15% of potential drug interactions were encountered. Nevertheless, the most common combination drug was ACEI with sulfonylurea in the study by Bob & Ines [7], whilst, in the current study, the combination of simvastatin and amlodipine contributed to the highest number of potential drug-drug interactions. Other significant potential drug interactions were the combination of antiplatelet and anticoagulant, and simvastatin and fenofibrate, as well as other combinations.

Studies have shown that simvastatin serum concentrations are significantly increased when used concurrently with amlodipine [27-29]. This is due to the fact that both simvastatin and amlodipine are substrates of CYP3A4. Subsequently, in 2011, the Food and Drug Administration (FDA) released a safety announcement on the dose limitation of 10 mg or 20 mg of simvastatin in order to reduce the risk of myopathy [30]. This alert has had a great impact on drug and dose selection in the management of T2DM patients with dyslipidemia as CCB was commonly prescribed (41%) to this group of patients. In the current study, it has been found that prescribers in the UMMC were not aware of the dose limitation issues, as about 10% of the sample subjects were given high dose simvastatin even though they were also prescribed CCB (amlodipine, diltiazem and verapamil) concurrently.

### Drug not taken/administered

Poor medication adherence was the second most common DRP (14.3%) found in this study [7,10,31]. This is in agreement with a study from the Netherlands, whereby 17.6% of T2DM patients were non-adherent to their medications [7]. This study also revealed that the non-adherence patients had significantly higher A1C values as compared to the compliant patients (9.7% versus 8.4%, p = 0.01). The significant relationship was in agreement with the study by Bob & Ines [7] (A1C of 9.4% versus 8.4%; p = 0.01). In addition, this study also showed a significant association between compliance issues and poor lipid control (p = 0.002). This critical finding indicated the importance of compliance to anti-diabetic and LLA drugs in T2DM patients with dyslipidemia in order to achieve better glycemic and lipid control.

More than 90% of poor medication compliance cases in this study were due to the patients forgetting to take medicines. This result is in accordance with a study by Lorenzo *et al*. [31]. Other reasons detected in this study were that patients had concerns over drugs due to their side-effects or the fact that they were unable to purchase medications from community pharmacy. Nevertheless, the findings were dependent on the honesty of the patient’s self-reporting compliance and the availability of data in the medical reports.

In contrast, Chan *et al.*[10] reported a much higher percentage of the same type of DRP in the geriatric population (35%). The explanation was most probably due to polypharmacy and a decrease in cognitive memory function in the geriatric patients [10].

### Causes associated with drug-related problems

Pharmacokinetic problems (code C1.4) were the most frequent causes associated with DRPs. Age-associated physiological changes leading to alteration of the pharmacokinetic and pharmacodynamic properties of drugs were one of the main contributors that caused DRPs [10]. In contrast, the current study (p = 0.662) found that there was no significant association between age and DRPs, which was in line with previous results [32].

In addition, drug-drug interactions that cause alterations in the pharmacokinetics of drugs also contributed to the high percentage of this DRP. For example, the concurrent use of simvastatin and amlodipine, which are both metabolized by the liver enzyme CYP3A4, causes a decrease in the metabolism rate of simvastatin [21,30]. Thus, healthcare providers should focus more attention on the alterations of pharmacokinetic properties which may be due to physiological factors or concurrent drug use. Changes in doses or shifting to alternative drugs may be required if the alteration of pharmacokinetics leads to significant adverse effect [21].

Under-dosing or overdosing in about 15% of T2DM patients with dyslipidemia was the second highest cause that was associated with DRP in this study. Non-optimal dosing in anti-diabetic and LLA drugs were the two most frequent causes of dosing problems (code C1.2) in this population of patients. A study by Bob & Ines [7] showed contrasting results, in which only 5.9% of the T2DM patients had dosing problems. The discrepancy may be explained by a more general group of T2DM patients who participated in a Pharmacy Diabetes Care Programme in Australia. Hence, this might not represent the most appropriate data of T2DM patients with dyslipidemia. From this study, it was shown that optimal dosing of both anti-diabetic and LLA can prevent DRP from occurring.

### Medications that cause drug-related problems

A quarter of patients had problems with antihypertensive agents in this study. This was followed by lipid-lowering agents (24.0%) and anti-diabetic agents (21.7%). This could be due to the fact that antihypertensive agents were prescribed to at least 80% of patients in this study. Combinations of antihypertensive agents are required in order to maintain the blood pressure level below 130/80 mmHg or 125/75 mmHg with proteinuria of more than 1 g/24 hours [1,18,19]. Thus, there was a higher possibility to develop DRPs secondary to the wide range of use of antihypertensive drugs. However, this study found that the use of antihypertensive agents was not significantly associated with an occurrence of DRP in T2DM patients with dyslipidemia (p = 0.465). This may be due to the small sample size of patients that had DRP secondary to antihypertensive agents in which random chance cannot be eliminated.

### Factors that were significantly associated with drug-related problems

#### Gender

In this study, a significant statistical difference was detected between gender and the occurrence of DRP. Male patients had a higher chance (96.4%) of having DRPs compared to female patients (86.5%). To date, there is a lack of studies focusing on the association of DRP with gender. However, a study by Babwah *et al.* in 2006 [33] reported that women who are unemployed have more time to attend clinic appointments and tend to be more compliant in terms of diet and medication when compared to men [33,34]. On the other hand, men who work and practice unhealthy habits, such as drinking alcohol and smoking, have a higher probability of having DRPs [33,34]. To date, there is a lack of evidence to suggest that biological factors associated with gender may affect the pharmacological treatment. Besides, in this study, the higher proportion of males compared to female patients may lead to the random chance of males having at least one DRP.

#### Renal impairment

Renal impairment showed a significant association with the occurrence of DRP in this study. A high percentage (about 98%) of diabetic dyslipidemia patients with renal impairment had at least one DRP. Evidences suggested that renal impairment patients require more pharmaceutical intervention as DRPs commonly occurred in all healthcare settings [9,35]. Similarly, this study found that DRPs of potential drug interaction (18.9%), untreated conditions such as anemia (10.8%) and poor medication compliance (10.8%) frequently occurred in diabetic dyslipidemia patients with chronic kidney disease.

The inappropriate use of renal risk drugs has been controversial in T2DM patients with dyslipidemia [35,36]. In long-term treatment, renal risk drugs, such as ACEI, calcium channel blockers and simvastatin, are in fact renoprotective drugs. These medications can decrease microalbuminuria, slow the progression to end stage renal failure and prevent cardiovascular events in high risk patients, especially T2DM patients with dyslipidemia [35,36]. A study has been proposed to examine the role of lipid control by statins in the prevention of nephropathy, as well as the additional effect of reducing proteinuria [37]. Therefore, it is recommended to use these drugs in caution with close monitoring for dosing adjustment due to the risk of causing DRPs in this population of patients.

#### Polypharmacy

The issue of polypharmacy is commonly reported as a risk factor that contributes to the occurrence of DRPs in different study subjects [32,38,39]. In agreement with a few studies, polypharmacy was shown to be significantly associated with DRP in T2DM patients with dyslipidemia, in which about 95% of patients with six or more drugs had at least one DRP [32,38,39]. Polypharmacy has been associated with problems such as poor medication adherence, potential drug-drug interactions and side-effects of drugs [38,39]. Patients with multiple drug classes of medicines often have a complex drug schedule. The frequent daily drug administration and different pill numbers for each medication may contribute to the poor medication adherence problem in these patients_38_. A recent study showed that DRPs secondary to polypharmacy will lead to the increased cost of treatment and hospitalization [32]. However, the under-treatment of disease by reducing the number of drugs may cause more serious consequences, especially in T2DM patients with dyslipidemia [39]. Hence, pharmacists play an important role in the optimization of drug treatment for the patient’s benefit.

#### Lipid control

The ability to achieve good control of lipid levels was shown to have a statistically significant association with the occurrence of DRP in T2DM patients with dyslipidemia. Poor lipid control in this study was defined as when at least one lipid parameter was not within the targeted range according to the ADA recommendations [1]. The current study showed that patients with poor lipid control were at a higher risk of developing DRPs (95.5%). This can be explained by the fact that patients with poor lipid control are at an increased risk of developing macrovascular complications, such as atherosclerosis, myocardial infarction, hypertension and stroke [1,3]. Theoretically, the development and progression of complications lead to more drugs being used in order to control the complications [4,38]. Thus, this increases the probability of DRP occurrence.

In addition, poor lipid control may induce the development and progression of nephropathy by releasing mediators, such as cytokines and reactive oxygen species, that cause injury to the glomerulus [37,40]. Hence, the progression of nephropathy secondary to poor lipid control may increase the probability of DRPs occurring. This suggestion is in line with the findings of the current study, in which nephropathy was significantly associated with DRP occurrence (p = 0.009). Therefore, a strict lipid control approach is important in T2DM patients with dyslipidemia. A poor lipid profile may enhance the development of microvascular and macrovascular complications that subsequently lead to the occurrence of DRPs.

The identification of underlying factors associated with DRPs may help in preventing and resolving DRPs in T2DM patients with dyslipidemia.

Therefore, the early identification of DRPs and factors associated with them may help to prevent and resolve DRPs in T2DM patients with dyslipidemia and thus enhance the most appropriate drug treatment and a more cost-effective pharmaceutical care.

### Study limitation

The retrospective nature of study design limits the information source in which the assessment of DRP was solely dependent on the medical records and biochemistry data from Laboratory Information System. Besides, lack of standard tools to recognise DRP in T2DM patients limits the comparison of DRPs with other studies.

## Conclusion

Potential drug interactions, poor medication adherence and the lack of health consciousness were the three most common factors found in this study. Factors associated with DRPs in T2DM patients with dyslipidemia were male gender, renal impairment, polypharmacy and poor lipid control.

## Competing interests

The authors declare that they have no competing interests.

## Authors’ contributions

HZH has made substantial contributions to conception and design, acquisition of data, analysis and interpretation of data and drafting the manuscript or revising it critically for important intellectual content. LCL has been involved in acquisition of data and analysis and interpretation of data. HZH and LCL have given final approval to the version to be published.

## Pre-publication history

The pre-publication history for this paper can be accessed here:

http://www.biomedcentral.com/1471-2458/13/1192/prepub
